# Full-Term Human Placental Macrophages Eliminate *Coxiella burnetii* Through an IFN-γ Autocrine Loop

**DOI:** 10.3389/fmicb.2019.02434

**Published:** 2019-10-29

**Authors:** Soraya Mezouar, Imene Benammar, Asma Boumaza, Aïssatou Bailo Diallo, Céline Chartier, Christophe Buffat, John Boudjarane, Philippe Halfon, Maria Katsogiannou, Jean-Louis Mege

**Affiliations:** ^1^Aix-Marseille Université, MEPHI, IRD, APHM, Marseille, France; ^2^IHU-Méditerranée Infection, Marseille, France; ^3^APHM, Biochemistry and Molecular Biology, Hôpital de la Conception, Marseille, France; ^4^INSERM U1251, Centre de Génétique Médicale MMG, APHM, Hôpital de la Timone, Aix-Marseille Université, Marseille, France; ^5^Laboratoire Alphabio, Hôpital Européen, Marseille, France; ^6^Department of Obstetrics and Gynecology, Hôpital Saint Joseph, Marseille, France; ^7^APHM, UF Immunologie, Marseille, France

**Keywords:** *Coxiella burnetii*, inflammation, interferon-γ, macrophages, multinuclear giant cells, placenta

## Abstract

The intracellular bacterium *Coxiella burnetii* is responsible for Q fever, an infectious disease that increases the risk of abortion, preterm labor, and stillbirth in pregnant women. It has been shown that *C. burnetii* replicates in BeWo trophoblast cell line and inhibits the activation and maturation of decidual dendritic cells. Although tissue macrophages are known to be targeted by *C. burnetii*, no studies have investigated the interplay between placental macrophages and *C. burnetii*. Here, CD14^+^ macrophages from 46 full-term placentas were isolated by positive selection. They consisted of a mixed population of maternal and fetal origin as shown by genotype analysis. We showed that *C. burnetii* organisms infected placental macrophages after 4 h. When these infected macrophages were incubated for an additional 9-day culture, they completely eliminated organisms as shown by quantitative PCR. The ability of placental macrophages to form multinucleated giant cells was not affected by *C. burnetii* infection. The transcriptional immune response of placental macrophages to *C. burnetii* was investigated using quantitative real-time RT-PCR *on* 8 inflammatory and 10 immunoregulatory genes. *C. burnetii* clearly induced an inflammatory profile. Interestingly, the production by placental macrophages of interferon-γ, a cytokine known to be involved in efficient immune responses, was dramatically increased in response to *C. burnetii*. In addition, a clear correlation between interferon-γ production and *C. burnetii* elimination was found, suggesting that macrophages from full-term placentas eliminate *C. burnetii* under the control of an autocrine production of interferon-γ.

## Introduction

The placenta is a unique chimeric organ made of fetal (chorion) and maternal (decidua) tissues at the materno-fetal interface. It is essential for nutritional exchanges and the immune tolerance of the fetus (Houser, 2012; Pinhal-Enfield et al., 2012). The latter is maintained by placental immune cells that prevent fetus rejection and protect it from infections. These immune cells mainly consist of natural killer (NK) cells and macrophages whereas T and B lymphocytes, dendritic cell,s and mast cells are less represented (Bulmer et al., 2010; Ben Amara et al., 2013; Gorvel et al., 2014; Mezouar et al., 2018, 2019c).

Placental macrophages represent 20–30% of the leukocytes found in all compartments of the placenta and their proportion increased throughout pregnancy (Bulmer et al., 2010). The phenotypic characterization of placental macrophages relies on the expression of CD68, a myeloid marker and CD14, a marker of circulating monocytes that is lacking in tissue macrophages other than placental macrophages (Ben Amara et al., 2013; Mezouar et al., 2019a). Placental macrophages include two populations of different origin. While Hofbauer cells are of fetal origin, decidual macrophages are of maternal origin (Pinhal-Enfield et al., 2012). These two populations are usually distinguished by their location within the placenta: Hofbauer cells are found in the fetal chorionic villi and decidual macrophages are found in decidua basalis (Pinhal-Enfield et al., 2012). Unfortunately, no specific phenotypic marker allows the distinction of these two populations. The role of placental macrophages in pregnancy is plural: they are involved in tissue remodeling, angiogenesis (Pongcharoen et al., 2007; Fong, 2008; Loegl et al., 2016), antigen presentation (Heikkinen et al., 2003, 2004), and maternal-fetal tolerance (Wang et al., 2018). They spontaneously form *in vitro* multinuclear giant cells (MGCs) even if the precise role of these MGCs remains to be elucidated (Ben Amara et al., 2013). It is well known that the activation status, also called polarization, of macrophages govern their biological activities: M1 macrophages are inflammatory and microbicidal whereas M2 macrophages are immunoregulatory and non-microbicidal. The M1/M2 status of macrophages is dependent on their microenvironment, especially on cytokines. Indeed, inflammatory cytokines such as tumor necrosis factor (TNF) and interferon (IFN)-γ induce a M1 polarization whereas immunoregulatory cytokines such as interleukin (IL)-10 induce a M2 polarization of macrophages (Benoit et al., 2008b; Mantovani et al., 2013). As reported for other macrophages, placental macrophages have been classified in either the M1 or M2 group. The M2 profile of placental macrophages is found at the beginning of pregnancy whereas the M1 profile is found at the end of pregnancy (Zhang et al., 2017). An inappropriate polarization of placental macrophages is associated with pregnancy complications such as abortion or miscarriage (Mezouar and Mege, 2018). Chorioamnionitis, a placental infection, interferes with placental macrophage polarization (Brown et al., 2014; Mezouar and Mege, 2018), and consists in an altered inflammatory response including decreased production of IL-10 (Ben Amara et al., 2013). For others, maintenance of the M2 polarization profile was observed (Joerink et al., 2011).

*Coxiella burnetii*, an intracellular gram-negative bacterium, is the causative agent of Q fever, a widespread zoonosis. *C. burnetii* infection leads to two major clinical forms. The primary infection is most often (60% of cases) asymptomatic and is usually spontaneously resolved. *C. burnetii* infection may by persist in the host for a minority of infected patients (less than 5%) leading to endocarditis or vascular infection (Raoult et al., 2005). *C. burnetii* infection of pregnant women is associated with specific problems (Carcopino et al., 2007) mainly due to the absence of clinical signs characteristic of the acute Q fever. The risk of pregnancy complications such as abortions and prematurity is high when the infection occurs during the first trimester. Malformations, stunting, or death *in utero* have also been reported (Eldin et al., 2017). Animal models of *C. burnetii* infection show that approximately 10^9^ bacteria are present in 1 g of placental tissue (Sobotta et al., 2017). An imbalance of cytokine production is also observed in pregnant goats infected by *C. burnetii* (Roest et al., 2012). In *C. burnetii*-infected women, it has been suggested that abortions are related to infected and inflammatory placentas (Shinar et al., 2012).

The cellular reservoirs of *C. burnetii* in placenta are likely diverse. When human BeWo trophoblast cell line is infected with *C. burnetii*, the bacteria replicate within acid phagosomes (Ben Amara et al., 2013). The analysis of the transcriptional signature of these trophoblasts reveals the up-modulation of genes associated with inflammation pathways (Ben Amara et al., 2010). We also showed that *C. burnetii* infects placental dendritic cells and prevents their maturation and likely their ability to present antigens to the adaptive immune system (Gorvel et al., 2014). To our knowledge, the effect of *C. burnetii* infection on the functional activity of placental macrophages is unknown. In this study, we showed that isolated CD14^+^ placental macrophages were able to eliminate *C. burnetii* within 9 days. The ability of these macrophages to spontaneously differentiate within MGCs was not affected by *C. burnetii* infection. In contrast, placental macrophages exhibited an inflammatory profile with an unexpected upregulated production of IFN-γ correlated with *C. burnetii* elimination.

## Materials and Methods

### Placenta Collection

The study was approved by the “Comité d’Ethique d’Aix-Marseille Université” (number 08-012). Forty-six full-term placentas were collected at the Gynecology-Obstetrics Department of the “Hôpital de la Conception” (Marseille, France) after informed consent of the mothers. Women were devoid of pathologies, with a mean age of 34 years (21–42 years), a gestational age of 39 weeks (36–42 weeks) with main vaginal delivery (44 vaginal deliveries *versus* 2 caesarean deliveries). The placentas did not show any lesions or inflammation by macroscopically observation.

### Bacteria

*Coxiella burnetii* Nine Mile strain (RSA496) was cultured as previously described (Ka et al., 2016). Briefly, L929 cells were infected for 8 days, sonicated, and centrifuged 10 min at 300 ×*g*. Bacteria were collected after centrifugation at 10,000 ×*g* for 10 min, then washed and stored at −80°C. The concentration and the bacterial viability were assessed using the LIVE/DEAD BacLight bacterial viability kit (Life Technologies). In some experiments, organisms were labeled with the membrane fluorescent marker 4-(4-(dihexadecylamino)styryl)-N-methylpyridinium *iodide* (DID, Thermo Fisher Scientific) for 20 min at 37°C in phosphate buffered saline (PBS).

### Isolation of Placental Macrophages

Placental macrophages were isolated as previously described (Mezouar et al., 2019a). Briefly, entire placenta tissue was digested in Hank’s Balanced Salt Solution (HBSS), DNase I 2.5 mM, and 2.5% trypsin (Life Technologies). Cell suspension was filtered through 100-μm pores and deposited on a Ficoll cushion and centrifuged at 700 ×*g* for 20 min to collect mononuclear cells. Placental macrophages were isolated using magnetic beads coated with anti-CD14 antibodies (Miltenyi Biotec). The purity of isolated CD14^+^ placental macrophages was assessed by flow cytometry and was higher than 98%.

### Flow Cytometry Phenotyping of Isolated CD14^+^ Placental Cells

The phenotype of isolated CD14^+^ placental macrophages was assessed as follows. Cells (1 × 10^6^ cells per assay) were stained using mice IgG1 anti-human CD14-APC (Allophycocyanin) and anti-human CD68-FITC (Fluorescein isothiocyanate) antibodies or isotype controls in PBS containing 5% Fetal Bovine Serum (FBS) for 30 min at 4°C. Cells were washed, fixed with 4% paraformaldehyde, centrifuged at 600 ×g for 5 min, and then diluted in PBS. Stained cells were then analyzed by flow cytometry (10,000 events/acquisition) using a BD FACS Canto II flow cytometer (BD biosciences). The results of flow cytometry were analyzed with FlowJo software.

### Placental Macrophage Genotyping

To evaluate the maternal or fetal origin of isolated macrophages, the placentas of male fetuses were genotyped using a commercial kit containing probes for detection of specific centromeric regions of X and Y chromosomes, and chromosome 18 was used as control, according to the manufacturer’s instructions (Fast Fish prenatal enumeration probe kit, Cytocell), as previously described (Huang et al., 2017). Briefly, after fixation using methanol and glacial acetic acid mix (3:1), placental macrophages were deposited onto a slide for fluorescence *in situ* hybridization including denaturation and hybridization of probes, and addition of Hoechst 33342 to DNA labeling. Slides were analyzed with a fluorescence microscope and the proportion of cells with maternal and fetal origin was determined.

### Formation of Multinuclear Giant Cells

Placental macrophages (2 × 10^5^ cells per assay) were incubated in 24 well plates containing glass coverslips in Dulbecco’s Modified Eagle Medium (DMEM)-F12 supplemented with 10% FBS, 100 U/ml penicillin, and 50 μg/ml streptomycin (Life Technologies) for 9 days, as previously described (Ben Amara et al., 2013). Every 3 days, the presence of MGCs was determined by DNA staining and labeling of filamentous actin (F-actin) with Hoechst 3342 and phalloidin-488 (Life Technologies), respectively. The formation of MGCs was then analyzed by confocal microscopy using an LSM 800 Airyscan confocal microscope (Zeiss). The number of MGCs was determined and the results expressed in percentage of cells presenting at least two nuclei.

### Lactate Dehydrogenase Assay

Isolated macrophages were incubated or not with *C. burnetii* (bacterium-to-cell ratio of 50:1) for 4 h and extensively washed to remove free bacteria (time designed as day 0). Cells were additionally cultured for 9 days, and the culture supernatants were collected at various time points. Lactate dehydrogenase release was quantified photometrically using Roche/Hitachi cobas c501 systems.

### Microbicidal Activity of Placental Macrophages

Isolated macrophages (2 × 10^6^ cells per assay) were cultured in DMEM-F12 supplemented with FBS and antibiotics. They were then incubated with *C. burnetii* (bacterium-to-cell ratio of 50:1) for 4 h and extensively washed to remove free bacteria (time designed as day 0). Placental macrophages were additionally cultured for 9 days and the uptake of bacteria was studied according two different approaches. First, macrophages were incubated with DID-labeled bacteria, stained with Hoechst 3342 and phalloidin-488, and the intracellular localization of bacteria was studied by confocal microscopy. Second, the uptake of bacteria was studied by qPCR. Briefly, DNA was extracted using a DNA Mini Kit (Qiagen), and infection was quantified using quantitative real time PCR (qPCR), as previously described (Ka et al., 2016). qPCR was performed with SYBR Green Fast Master mix (Roche Diagnostics) and the CXF Touch Real-Time PCR Detection System (Bio-Rad) using F (5′-GCACTATTTTTAGCCG-GAACCTT-3′) and R (5′-TTGAGGAGAAAA-ACTGGATTGAGA-3′) primers that amplified a 225-bp fragment of the *C. burnetii com1* gene. A standard curve was generated using serial dilutions from a known concentration of *C. burnetii* DNA.

### Inflammatory Response of Placental Macrophages

Two different approaches were used to study the inflammatory response of placental macrophages. First, the transcriptional response of 8 M1 genes and 10 M2 genes was studied by real-time quantitative PCR (qRT-PCR). For that purpose, placental macrophages (3 × 10^5^ cells per assay) were incubated with *C. burnetii* (bacterium-to-cell ratio of 50:1), or 1 μg/ml lipopolysaccharide (LPS, Sigma-Aldrich) as control, for 6 h. Total RNA was extracted using RNeasy Mini Kit (Qiagen) and DNAse I treatment to eliminate DNA contaminants, as previously described (Mezouar et al., 2019b). The quantity and the quality of RNA were evaluated using a Nanodrop spectrophotometer (Nanodrop Technologies). Reverse transcription of isolated RNA was performed using a Moloney murine leukemia virus-reverse transcriptase kit and oligo(dT) primers (Life Technologies). qRT-PCR was performed using SYBR Green Fast Master mix (Roche Diagnostics) and a CFX Touch real-time PCR Detection System (Bio-Rad) using specific primers (Ben Amara et al., 2013) listed in Table 1. The results were normalized using the housekeeping *actb* gene encoding β-actin endogenous control and were expressed as relative quantity (RQ) using the following formula: RQ = 2^−ΔCt^, where ΔCt = (Ct_Target_ − Ct_Actin_). The threshold cycle (Ct) was defined as the number of cycles required to detect the fluorescent signal. Data were analyzed using Clustvis software.

**Table 1:** List of genes associated with macrophage polarization.

Gene symbol	Forward primer (5′-3′)	Reverse primer (5′-3′)
β-actin	GGAAATCGTGCGTGACATTA	AGGAGGAAGGCTGGAAGAG
**M1 genes**		
*CCL2*	GCTGGAGAGCTACAAGAGGATCA	TCTCTCTTGAGCTTGGTGACAAAA
*NOS2*	TTGCAAGCTGATGGTCAAGATC	CAACCCGAGCTCCTGGAA
*EDN1*	CCTCCATCCCCCATACTAAATC	GTCTCCAAAAATCAAGGACAGG
*HESX1*	GCTCGGGGAAAACAAACC	TTCTTCTGGCATTGGGTGA
*IDO1*	TCATCTCACAGACCACAAGTCA	CAAAATAGGAGGCAGTTCCAGT
*TNFSF10*	GAAAATAATCCCCACACGCTAC	GTCACTCTCTCCACCCTCACA
*IL15RA*	ATCTTCCGTCCCTCATCCTAAC	CTCAGCATCTCTCCCACCTTT
*CXCL9*	ACACTTGCGGATATTCTGGACT	GGGAGATGGTGTGTAATTGAT
*IL2RA*	GTTGAAGAGGAAGGGCAAAAC	ACTGGGAAGTTGGAATGAGATG
*TNF*	CATCTATCTGGGAGGGGTCTTC	AGGAGGGGGTAATAAAGGGATT
**M2 genes**		
*ALOX15*	AACTTCCACCAGGCTTCTCTC	GGGGGCTGAAATAACCAAAG
*CCL13*	GAGCAGAGAGGCAAAGAAACA	ATGTGAAGCAGCAAGTAGATGG
*CCL23*	CATCTCCTACACCCCACGAA	CATTCTCACGCAAACCTGAACT
*CLEC4F*	GGCATTTCTGGTAGAGTTCACA	ATACTTTCTGAGTGGGCAGGA
*CHN2*	AGAACTGTGGCTGGAAAATGAG	GTGGTTGTCGTTGTGTGTGAG
*CTSC*	GAGGTTGTGTCTTGTAGCCAGT	CCCCTTTTTGTAGTGGAGGAAG
*FN1*	ACACCTGGAGCAAGAAGGATAA	CCACAGAGTAGACCACACCACT
*HRH1*	ACTTGGAGGTGGTATGTGCTG	CTCAGGGCTTGCTTCTTGTAGT
*SLC4A7*	CCCTCAAAACAGTCCTCCTTCT	TTTCCTCATTCTTCTGCTCCTC
*CD209*	GGATACAAGAGCTTAGCAGGGTG	GCGTGAAGGAGAGGAGTTGC

Second, the release of IFN-γ by placental macrophages was studied using specific immunoassay kits (R & D Systems). Briefly, placental macrophages (3 × 10^5^ cells) were stimulated or not with *C. burnetii* (bacterium-to-cell ratio of 50:1) for 6 h. The supernatants were centrifuged at 1,000 × g for 10 min and frozen at −80°C. In some experiments, placental macrophages (1 × 10^6^ cells) were incubated or not with *C. burnetii* for 4 h and, after washing to remove free bacteria (time designed as day 0), they were cultured for 9 days. Every 3 days, supernatants were collected and the release of IFN-γ was quantified. The sensitivity of the assays was 1.0 pg/ml.

### Statistical Analysis

Statistical analysis was performed using GraphPad Prism software version 7.0a (GraphPad Software, La Jolla California USA, www.graphpad.com). *In vitro* data were analyzed using the Mann-Whitney *t* test, one-way ANOVA, or ANOVA Kruskal-Wallis test. Correlations were evaluated using the non-parametric Spearman test. The results are presented as the mean score of at least three independent experiments, and *p* < 0.05 was considered statistically significant.

## Results

### Characterization of Placental Macrophages

**Figure 1:**
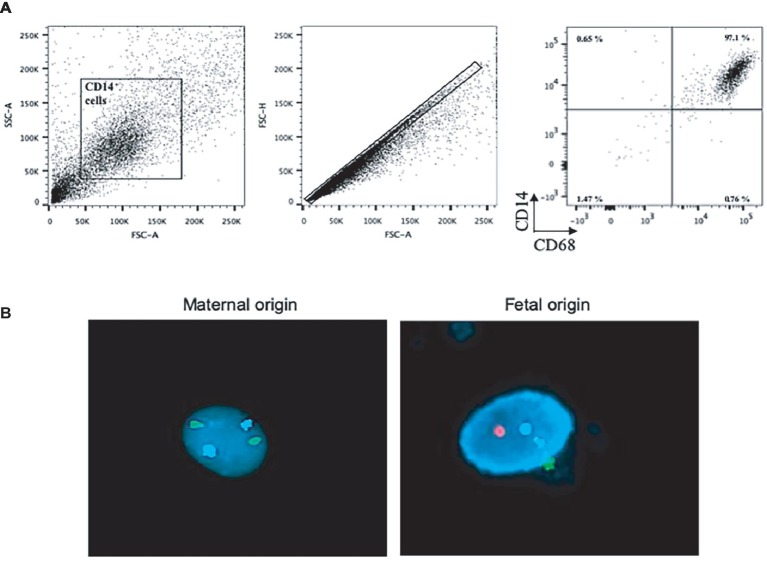
Characterization of placental macrophages. **(A)** Placental CD14^+^ cells from 20 different placentas were analyzed for the expression of CD14 and CD68 by flow cytometry. Representative dot-blots are shown. **(B)** Placental macrophages from the placentas of male fetuses were analyzed for the expression of X, Y, and 18 chromosomes. Representative pictures show the X chromosome (in green), the Y chromosome (in red), and the chromosome 18 (nucleus in blue, used as control).

To ensure that placental CD14^+^ cells from healthy donors were macrophages distinct from maternal circulating monocytes, we used flow cytometry to assess the expression of CD68, a canonical macrophage marker, by CD14^+^ cells. CD14^+^ cells also expressed CD68 (Figure 1A), demonstrating that they are placental macrophages. Then, we evaluated the fetal or maternal origin of isolated CD14^+^ placental macrophages using fluorescence *in situ* hybridization technique and fluorescent probes targeting the X and Y chromosomes (Figure 1B). The study of five different placentas of male fetuses showed that approximately 30% of macrophages expressed XX (maternal) chromosomes and 70% expressed XY (fetal) chromosomes. These findings showed that placental CD14^+^ macrophages from healthy donors were a mixed population of Hofbauer cells and decidual macrophages.

### *C. burnetii* Infection of Placental Macrophages

**Figure 2:**
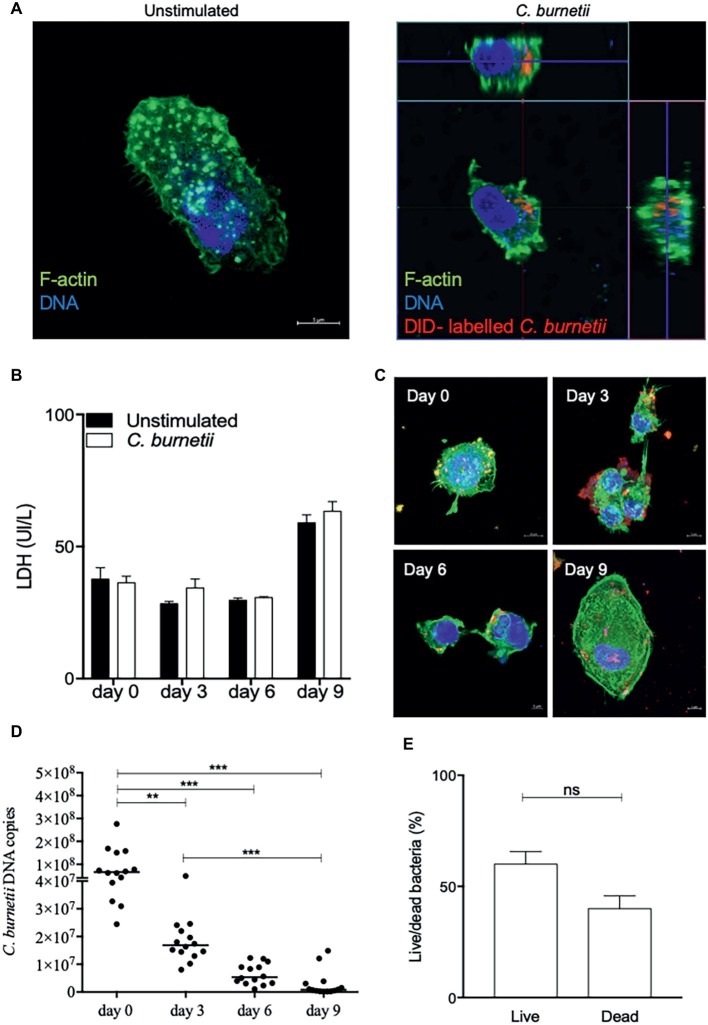
Microbicidal activity of placental macrophages. Placental CD14^+^ macrophages (2 × 10^6^ cells per assay) were infected or not by *C. burnetii* (bacterium-to-cell ratio of 50:1) for 4 h. **(A)** Confocal pictures show placental macrophage infected of not by DID-labeled bacteria. Infected macrophages were illustrated by the sections at the top and the right of the image was bacteria indicated in red, F-actin labeled with phalloidin-488 in green, and nucleus stained with Hoechst 3342 in blue. **(B)** Placental macrophages were incubated with *C. burnetii* for 4 h (day 0), then washed to eliminate free bacteria and incubated for 9 days. Lactate dehydrogenase (LDH) quantification was performed at each time of kinetic time of infection (*n* = 4 placentas). **(C)** Pictures of placental macrophages infected by *C. burnetii* at 0-, 3-, 6- and 9-day post-infection. Bacteria are indicated in red, F-actin labeled with phalloidin-488 in green and nucleus stained with Hoechst 3342 in blue. **(D)** Every 3 days, the number of bacterial DNA copies was evaluated by qPCR (*n* = 14 placentas). **p ≤ 0.01 and ***p ≤ 0.001. **(E)** Percentage of live/dead bacteria number was evaluated per macrophages at each time (ns = not significant).

Isolated CD14^+^ placental macrophages were infected or not with *C. burnetii* (bacterium-to-cell ratio of 50:1) for 4 h (Figure 2A) and cells were washed to remove unbound bacteria (this time was defined as day 0). Then, every 3 days of infection, the lactate dehydrogenase (LDH) release was quantified. No significant differences were observed between infected and resting placental macrophages (Figure 2B). The uptake, replication, or elimination of organisms by macrophages was observed (Figure 2C), and quantified by evaluation of the number of bacterial DNA copies using qPCR targeting the *com1* gene (Figure 2D). As a single *com1* gene is found per bacterium, it was easy to deduce that about 30 bacteria infected placental macrophages at day 0. A significant and steady decrease in the number of bacterial DNA copies was observed. At day 3, the number of bacterial DNA copies (1.6 ± 0.9 × 10^7^) significantly (*p* = 0.0049) decreased (75%) to reach 8.11% of initial value at day 6. At day 9, the number of bacterial copies represented only 1.22% of the number found at day 0 (Figure 2D) with as many live bacteria as dead (Figure 2E). Taken together, these results provide evidence that placental macrophages were able to internalize *C. burnetii* organisms and that they had a microbicidal effect on these bacteria.

### *C. burnetii* Infection and Multinuclear Giant Cell Formation

**Figure 3:**
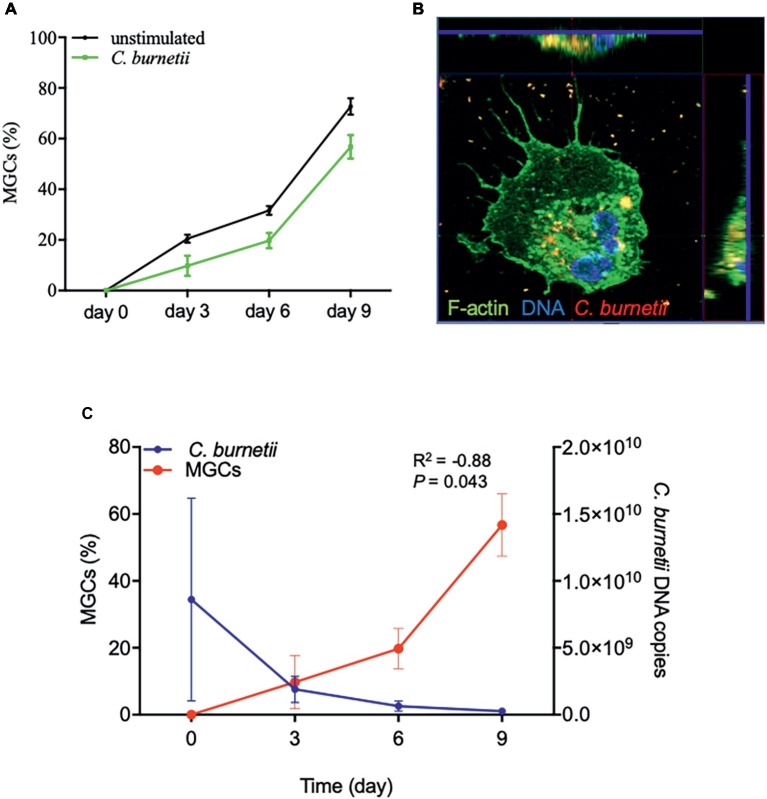
MGC formation and *C. burnetii* infection. Isolated placental macrophages (2 × 10^5^ cells per assay) from eight different placentas were stimulated by *C. burnetii* (bacterium-to-cell ratio of 50:1) for 4 h, then washed to eliminate unbound bacteria and cultivated for 9 days. **(A)** The time course of the number of MGCs was determined by optical determination and the results expressed in percentage of cells presenting at least two nuclei. **(B)** A representative confocal picture is shown after 3 days with F-actin labeled with phalloidin-488 in green, nucleus stained with Hoechst 3342 in blue, and bacteria in red. Intracellular bacteria were observed in the sections found at the top and the right of the image. **(C)** The analyses of MGC formation and *C. burnetii* survival performed using four placentas are correlated (*R*^2^ = −0.88, *p* = 0.043).

Because placental macrophages spontaneously form MGCs after culture, we wondered if *C. burnetii* interfered with MGC formation. Placental macrophages were infected with *C. burnetii* (bacterium-to-cell ratio of 50:1) for 4 h (day 0), washed to discard unbound bacteria and the formation of MGCs was quantified from day 0 to day 9. MGC formation was similar in uninfected and *C. burnetii*-infected macrophages (Figure 3A), demonstrating that *C. burnetii* did not prevent MGC formation. We then assessed the ability of these MGCs to ingest *C. burnetii*. MGCs formed after 3 days of culture were able to ingest bacteria as demonstrated by confocal imaging (Figure 3B). Interestingly, the formation of MGCs and *C. burnetii* DNA copies were significantly correlated (*R*^2^ = −0.88, *p* = 0.043) (Figure 3C), suggesting that MGCs derived from placental macrophages were involved in *C. burnetii* elimination.

### Molecular Mechanism of *C. burnetii* Elimination

**Figure 4:**
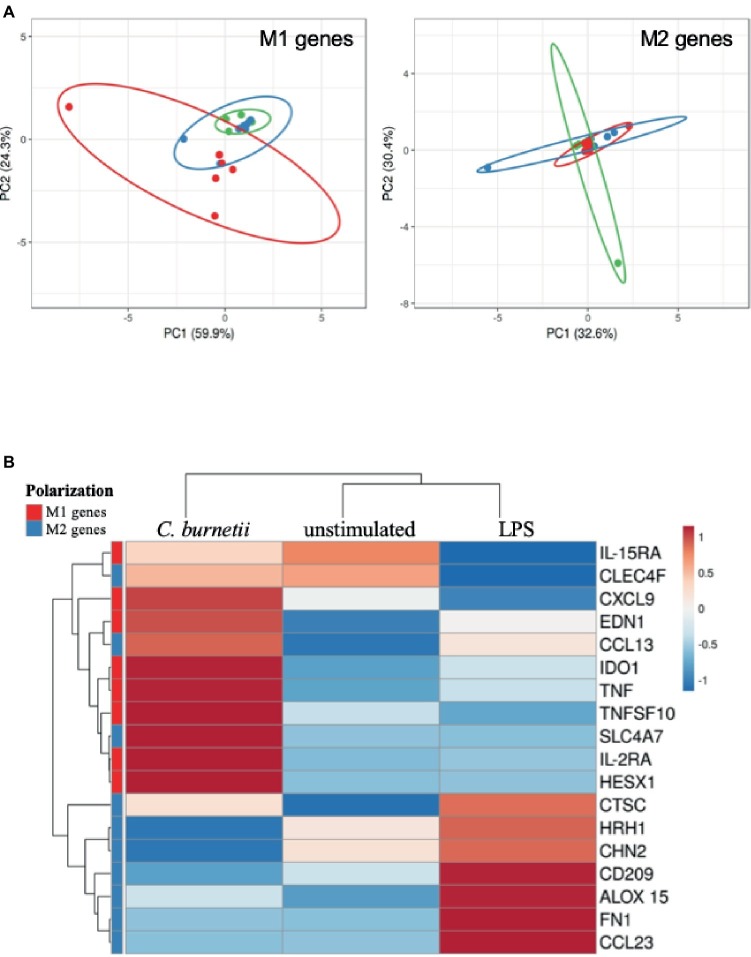
Polarization profile of placental macrophages stimulated by *C. burnetii.* Placental macrophages (1 × 10^6^ cells per assay) were incubated with *C. burnetii* (bacterium-to-cell ratio of 50:1) or 1 μg/ml LPS for 6 h, and their M1/M2 transcriptional response was analyzed by qRT-PCR. **(A)** A principal component analysis showed the repartition of unstimulated placental macrophages (in green, six placentas), *C. burnetii-*stimulated (in red, seven placentas), and LPS-stimulated macrophages (in blue, seven placentas) according to the expression of M1 genes (left panel) and M2 genes (right panel). **(B)** A heat-map analysis showed the modulation of the relative quantity expression of M1 genes (in red) and M2 genes (in blue) when placental macrophages were unstimulated or stimulated by *C. burnetii* or LPS.

To understand how bacteria were eliminated by placental macrophages, we investigated their transcriptional response to *C. burnetii*, and LPS as control, after 6 h of incubation. First, the expression of 8 M1-related genes including *IL-15RA*, *CXCL9*, *EDN1*, *IDO1*, *TNF*, *TNFSF10*, *IL-2RA*, and *HESXI* and 10 M2-associated genes including *CLEC4F*, *CCL13*, *SCL4A7*, *CTSC*, *HRH1*, *CHN2*, *CD209*, *ALOX15*, *FN1,* and *CCL23* was evaluated by qRT-PCR. Using a principal component analysis approach of M1 genes, we found that unstimulated and LPS-stimulated macrophages were associated whereas *C. burnetii*-stimulated macrophages were in an eccentric position. In contrast, the expression of M2 genes was strongly interrelated in unstimulated, LPS- and *C. burnetii*-stimulated cells (Figure 4A). A hierarchical clustering approach showed that modulated genes of placental macrophages stimulated by *C. burnetii* formed a cluster distinct from those of unstimulated and LPS-stimulated macrophages (Figure 4B). M1 genes are found in a different cluster than that of M2 genes, even if they included the *CLEC4F*, *CCL13,* and *SCLC4A7* M2-related genes. Interestingly, *C. burnetii* induced the up-modulation of all M1 genes compared to unstimulated and LPS-stimulated cells. Contrary to *C. burnetii*, LPS stimulation of placental macrophages led to the up-modulation of M2 genes. Taken together, these results demonstrated that *C. burnetii*, and not LPS, induced a M1-type transcriptional profile.

**Figure 5:**
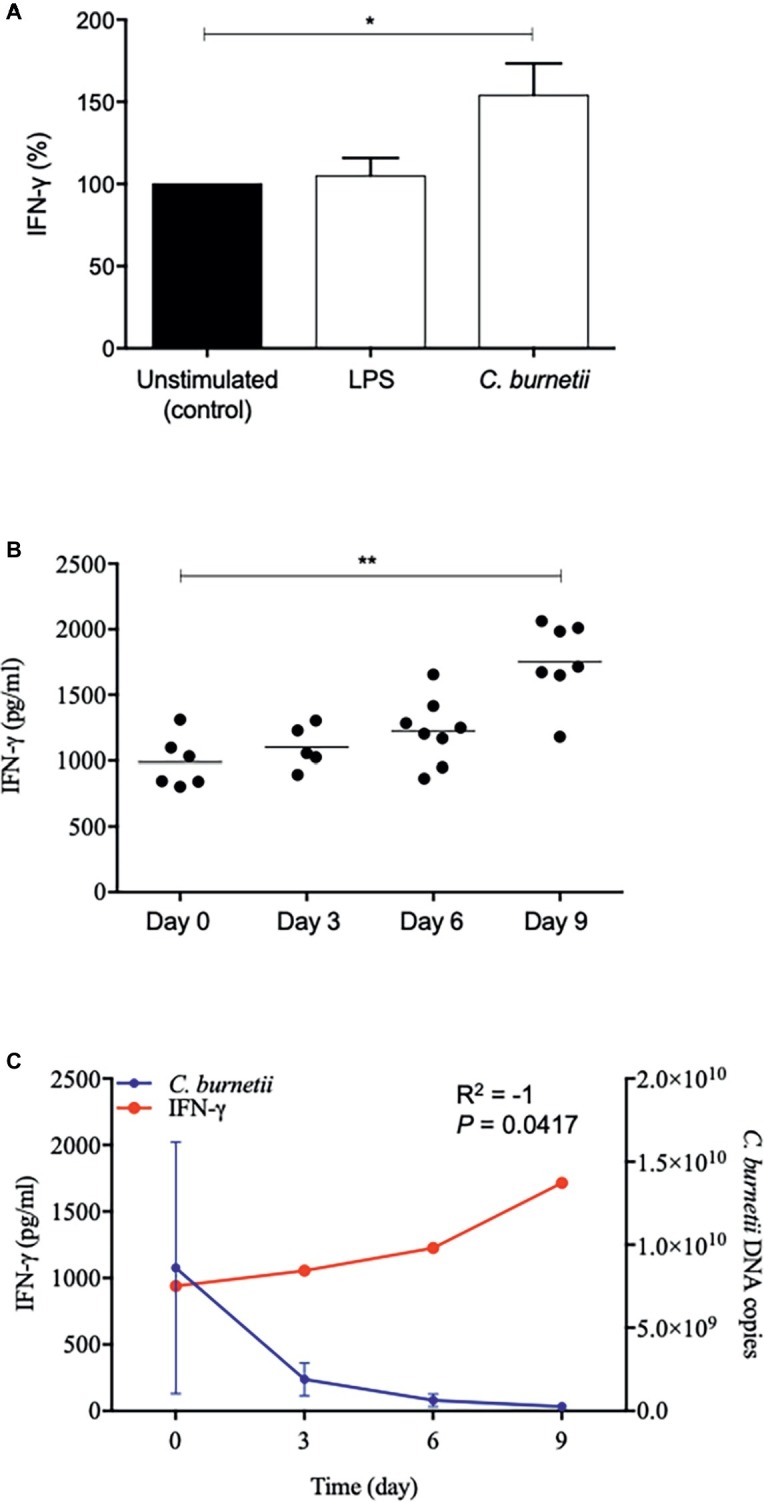
IFN-γ release and *C. burnetii* infection. **(A** and **B)** Placental macrophages (1 × 10^6^ cells per assay) were incubated or not with *C. burnetii* (bacterium-to-cell ratio of 50:1) or 1 μg/ml LPS for 4 h (day 0) and then washed to eliminate free bacteria. The release of IFN-γ was quantified at 0-, 3-, 6-, and 9-day post-infection or unstimulated cells as control when six placentas were used. **(C)** The release of IFN-γ by placental macrophages was correlated with *C. burnetii* survival (*R*^2^ = −1, *p* = 0.0417) when four placentas were used. *p ≤ 0.05 and **p ≤ 0.01.

Second, we investigated IFN-γ release by placental macrophages stimulated by *C. burnetii*. Placental macrophages stimulated by *C. burnetii* induced a significant increase of IFN-γ release compared to unstimulated cells (*p* = 0.038, One-way ANOVA) (Figure 5A). Then, every 3 days of infection we showed a gradual increase of IFN-γ release over time (*p* = 0.0041, ANOVA Kruskal-Wallis test) (Figure 5B), suggesting a key role for this cytokine in cell response to bacterial infection. Finally, while investigating a relationship between IFN-γ release and *C. burnetii* survival, we showed a significant correlation between these two data (**R*^2^* = −1, *p* = 0.0417, non-parametric Spearman test) (Figure 5C). Collectively, these results suggest that IFN-γ production was correlated with *C. burnetii* elimination.

## Discussion

In the present study, we first characterized CD14^+^ cells isolated from full-term placentas. We found that more than 97% of CD14^+^ placental cells expressed CD68, a marker associated with macrophage differentiation confirming that CD14^+^ placental cells were macrophages, not circulating monocytes (Pinhal-Enfield et al., 2012). Next, we quantified fetal and maternal cells in placental macrophages by genotyping. We found a mixed cell population with maternal and fetal origins, even if the proportion of fetal macrophages seemed to be higher than that of maternal cells. In previous studies, the origin of placental macrophages (Hofbauer cells *versus* decidual macrophages) was based on the location of these cells within the placenta tissue (Reyes and Golos, 2018). These findings highlight the phenotype and origin of placental macrophage populations.

Here, we provided evidence that placental macrophages belong to the target cells for *C. burnetii,* as determined by the efficiency of bacterial uptake. This finding amplifies our previous observations that BeWo trophoblast cell line (Ben Amara et al., 2010) and decidual dendritic cells (Gorvel et al., 2014) are able to internalize *C. burnetii* whereas placental mast cells exhibit a powerful extracellular antimicrobial mechanism against the bacterium (Mezouar et al., 2019c,d). In contrast to BeWo trophoblast cell line, *C. burnetii* was eliminated by macrophages from full-term placentas. The use of macrophages from full-term placentas is consistent with the observation that the risk of fetal infection by *C. burnetii* is higher during the first and second trimesters than during the third trimester (Eldin et al., 2017).

*C. burnetii* did not prevent the spontaneous formation of MGCs, a peculiar property of placental macrophages (Ben Amara et al., 2013; Belhareth et al., 2018). This is distinct from infectious placental pathologies such as chorioamnionitis in which MGC formation is prevented (Ben Amara et al., 2013). Additionally, MGC formation and *C. burnetii* elimination were correlated, suggesting a microbicidal role of these cells derived from placental macrophages. This hypothesis is supported by the observation that MGCs from other origins can play an anti-microbial role (Enelow et al., 1992; Hernandez-Pando et al., 2000).

It is well known that the functional properties of macrophages including microbicidal activity are controlled by their microenvironment. We found that *C. burnetii* induced a transcriptional program consisting of M1-related genes. Surprisingly, LPS, a powerful M1 agonist of tissue macrophages, rather induced an M2 program, demonstrating that the placental microenvironment governs the inflammatory or the immunoregulatory activity of placental macrophages. This is distinct from our previous results with monocyte-derived macrophages that express an atypical M2 program in response to *C. burnetii* associated with bacterial persistence (Benoit et al., 2008a). We suggest that *C. burnetii* induces a M1 program in placental macrophages that, in turn, plays a role in the elimination of the bacteria.

Interestingly, we showed that two members of the TNF family, including TNF and TNFSF10, were up-modulated by *C. burnetii* infection compared to LPS stimulation. A large variety of anti-microbial responses have been attributed to genes of the TNF family such as direct killing of infected cells, inhibition of intracellular pathogen or induction of apoptosis (Rahman and McFadden, 2006). Here, we found an absence of cell apoptosis and a strong anti-microbial response. The anti-microbial role of TNF has been previously reported using mice model. Indeed, during *C. burnetii* infection TNF deficient mice presented a defect of infection control as for other pathogens such as *Listeria monocytogenes* or *Mycobacterium tuberculosis* (Rahman and McFadden, 2006; Andoh et al., 2007). Interestingly, during pregnancy an excess of TNF production by inflammatory immune cells leads to the abrogation of fetus tolerance and its rejection as observed in pregnant women with *C. burnetii* infection (Chabtini et al., 2013; Eldin et al., 2017). In contrast, other inflammatory cytokines such as IL-1β are not specifically modulated by *C. burnetii* (data not shown). All together these findings suggested that further investigations are needed to clarify the role of genes for TNF family in the placental response during *C. burnetii* infection.

We also found that *C. burnetii* stimulated the release of large amounts of IFN-γ by placental macrophages. IFN-γ is known to be produced by innate lymphoid cells, NK and Th1 cells but tissue macrophages are generally considered as poor producers of IFN-γ. The placental context plays likely an important role in the ability of placental cells to produce IFN-γ. Indeed, trophoblasts produce high levels of type III interferons (Aboagye-Mathiesen et al., 1994; Lee et al., 2001) that are likely to be involved in pregnancy. The role of the IFN-γ produced by placental macrophages remains elusive. Interestingly, the release of IFN-γ by placental macrophages and their microbicidal activity were significantly correlated, suggesting that an autocrine loop could lead to their microbicidal activity. Additionally, IFN-γ was found involved in the increased expression of IDO1 leading to the deprivation of tryptophan, as essential amino acid for bacteria (Nelp et al., 2018). Interestingly, we found an up modulation of IDO1 in *C. burnetii* infected placental macrophages. Indirectly it has been previously reported that this process lead the inhibition of bacterial growth (Pantoja et al., 2000). This result is reminiscent of the observations that the co-culture of decidual macrophages and NK cells increases the production of IFN-γ, which contributes to control the infection of macrophages with HIV (Quillay et al., 2016).

In conclusion, our results showed that macrophages from full-term placentas were resistant to *C. burnetii* infection; such resistance may be related to an autocrine effect of IFN-γ. This study provides new insight into the pathophysiology of Q fever disease at the placenta level and suggests the microbicidal role of MGCs.

## Data Availability Statement

All datasets generated for this study are included in the manuscript/supplementary files.

## Ethics Statement

The studies involving human participants were reviewed and approved by “Comité d’Ethique d’Aix-Marseille Université” (number 08-012). The patients/participants provided their written informed consent to participate in this study.

## Author Contributions

SM and J-LM conceived and designed the experiments. SM, IB, AB, and AD performed experiments. CC, JB, and CB performed flow cytometry, genotype, and LDH assay experiments, respectively. SM, IB, AB, and AD analyzed the data. SM, PH, MK, and J-LM supervised the study. SM and J-LM wrote the paper.

### Conflict of Interest

The authors declare that the research was conducted in the absence of any commercial or financial relationships that could be construed as a potential conflict of interest.
